# Population-Based Study of the Association between Urbanization and Kawasaki Disease in Taiwan

**DOI:** 10.1155/2013/169365

**Published:** 2013-06-23

**Authors:** Wei-Pin Chang, Shyh-Jong Wu, Wei-Chiao Chang, Ho-Chang Kuo

**Affiliations:** ^1^Department of Healthcare Management, Yuanpei University, Hsinchu 300, Taiwan; ^2^Department of Medical Laboratory Science and Biotechnology, Kaohsiung Medical University, Kaohsiung, Taiwan; ^3^Department of Clinical Pharmacy, School of Pharmacy, Taipei Medical University, Taipei 110, Taiwan; ^4^Department of Pharmacy, Taipei Medical University-Wanfang Hospital, Taipei 110, Taiwan; ^5^Institute of Master Program for Clinical Pharmacogenomics and Pharmacoproteomics, School of Pharmacy, Taipei Medical University, Taipei 110, Taiwan; ^6^Cancer Center, Kaohsiung Medical University Hospital, Kaohsiung Medical University, Kaohsiung 807, Taiwan; ^7^Department of Pediatrics, Kaohsiung Chang Gung Memorial Hospital and Chang Gung University College of Medicine, Kaohsiung 807, Taiwan

## Abstract

*Background.* It is unclear if the prevalence of Kawasaki disease (KD) correlates with the degree of urbanization. We hypothesized that the prevalence of KD is more pronounced in urban versus rural environments. *Methods.* The National Health Insurance (NHI) program was implemented in Taiwan in 1995 and covers most of the population (>99%). We used the NHI database to investigate the epidemiological features of KD. A total of 115 diagnosed patients with KD from 1997 to 2010 were included, together with 1,150 matched controls without KD. Chi-square analyses were performed to investigate the difference between modern city and rural environments. *Results.* Of the 1265 sampled subjects (claims data from 1,000,000 random subjects), the mean age of the KD study group and control group was 2.08 ± 1.66 and 2.08 ± 1.64 years, respectively. After matching for age, sex, and same index date, no statistically significant differences in urbanization level and geographical location of the patients' residence were observed. *Conclusion.* Urbanization did not appear to be an important effect modifier of Kawasaki disease in Taiwan.

## 1. Introduction

Kawasaki disease (KD) involves multisystemic vasculitis of unknown cause. KD is a global disease and mainly affects children less than 5 years old, with the highest incidence reported in Asia, especially in Japan, Korea, and Taiwan [1, 2]. The major clinical characteristics of KD are prolonged fever, bilateral nonpurulent conjunctivitis, diffuse mucosal inflammation, polymorphous skin rashes, indurative angioedema of the hands and feet, and nonsuppurative cervical lymphadenopathy [3–5]. The most serious complications of KD are coronary artery dilation (CAD) such as myocardial infarction, coronary artery dilation, coronary fistula, or coronary artery aneurysm [3–5]. Both genetic factors and environmental factors have been considered as playing an important role in the prevalence of KD. 

Brosius et al. [6] reported that the incidence of atopic dermatitis among children with Kawasaki disease is 9 times higher than that of controls. Webster et al. [7] reported that KD patients were more likely to have been admitted at least once with asthma/allergy than controls. Kuo et al. reported that T-helper (Th) type 2 immune response was elevated in the acute stage of KD, including eosinophils [8], IL-4, IL-5 [9], and eotaxin, than in the age-matched control then subsided within a normal range in the convalescent stage. The results imply a potential association between allergic diseases and KD. 

Several lines of evidence from epidemiological studies indicated that the increasing prevalence of allergic diseases such as asthma in developing countries may be due to the transition from traditional/rural to modern/urban societies. Rural areas tend to have lower asthma prevalence [10]. In addition, several factors related to urbanization such as environmental pollution, household exposure to allergens, and diet have been identified as risk markers for allergic diseases [11]. Indeed, the prevalence of allergic diseases is increasing with increasing levels of urbanization. However, the correlation between the prevalence of KD and urbanization is still unclear. In Taiwan, national monitoring of KD was started in March 1995 and was based on data collected from Taiwan's national health insurance (NHI) database. Today, the NHI covers more than 99% of Taiwan's population [12]. The entire database might be the largest such health insurance database currently available in the world. Therefore, this database can serve as a rich source of nationwide data. In this study, we used data collected from Taiwan's NHI health insurance database to analyze the association between KD and urbanization.

## 2. Methods

### 2.1. Study Database

A compulsory National Health Insurance (NHI) mechanism was implemented in Taiwan in 1995. The Taiwan NHI database includes data on complete outpatient visits, hospital admissions, prescriptions, disease, and vital status of 99% of the population of Taiwan, which includes 23 million beneficiaries. To help researchers perform studies of issues relevant to the NHI program, the Taiwan National Health Research Institute (NHRI) created and released the LHID2005 dataset to the public for research purposes. The LHID2005 dataset included registration and medical claims for 1,000,000 randomly sampled individuals from among the 23 million beneficiaries registered in the NHI program in 2005. The NHRI claimed that there were no statistically significant differences in age, sex, and health care cost between the one million randomly sampled individuals and the 23 million enrolled beneficiaries. This dataset contained deidentified secondary data; therefore, information could not be used to identify any one individual. As the NHRI had addressed the confidentiality assurance issue, a full review of this study was waived by the Institutional Review Board.

For LHID2005 dataset completeness and accuracy, data were audited by the Department of Health and the Bureau of the NHI. The LHID 2005 dataset was subsequently used extensively in many epidemiological studies.

### 2.2. Measures of Urbanization

The 359 communities in Taiwan were stratified into 7 urbanization categories according to the standard published by the Taiwanese National Health Research Institute (NHRI), with code 1 indicating most urbanized and code 7 indicating least urbanized. The NHRI used cluster analysis to split them into urbanization groups, the results of which identified 7 clusters based on the Taiwan census data of the year 2000. These classification criteria included population density (persons per km^2^), percentage of people with college-level education or higher, percentage of elderly people (older than 65 years), percentage of agricultural workers in the population, and number of physicians per 100,000 population [13]. In order to distinguish the geographical locations, the subjects were classified into four regions in Taiwan: north, center, south, and east.

### 2.3. Case Definition and Control Selection

We identified 115 patients who were initially hospitalized with a principal diagnosis of Kawasaki disease (ICD-9-CM code: 446.1) between January 1, 1997 and December 31, 2010. We assigned those subjects who received intravenous immune globulin (IVIG) and adopted the first hospitalization for treatment of Kawasaki disease during this period as the index date. So each KD patient was identified by admission as well as IVIG treatment to decrease selection bias. In our control study, each case was individually matched to 10 randomly selected controls on the basis of age, sex, and index date. Control patients were assigned the same index date as their corresponding case patients.

### 2.4. Statistical Analysis

All data processing and statistical analyses were performed with Statistical Package for Social Science (SPSS) Version 18 software (SPSS Inc). We used the chi-square test for independence to compare the differences between cases and controls in terms of geographical location, and for the urbanization level of the patient's residence (1 being the most urbanized and 7 being the least), we defined statistical significance as a two-sided *P* value of less than 0.05.

## 3. Results

**Figure 1:**
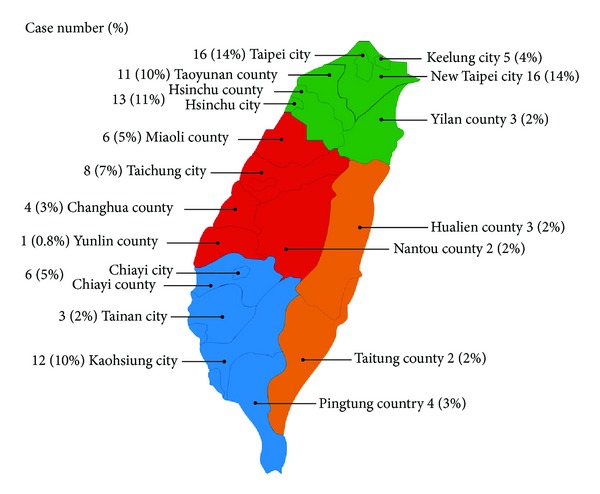
The map of Taiwan shows 19 counties/cities on the main island divided into 4 areas (north in green, center in red, south in blue, and east in orange).

As shown in Table 1, urbanization was divided into seven levels based on population density. In the 1265 sampled subjects (data from 1,000,000 random subjects), the mean age of the Kawasaki disease study group and control group was 2.08 ± 1.66 and 2.08 ± 1.64 years, respectively. After matching for age, sex, and same index date, as shown in Table 1, we found that most cases resided in levels of higher urbanization and resided in northern Taiwan (green color in Figure 1). After statistical computing, Table 2 demonstrated that there were no statistically significant differences in urbanization level (levels 1–7) and geographical location (north, center, south, and east) of the patient's residence between the study group and comparison group. The results in Figure 1 also showed that most cases are in northern Taiwan, but there were no statistical differences between the region level and KD.

## 4. Discussion

 Allergic diseases showed association with urbanization. Eriksson et al. [14] found a lifelong protective effect of childhood farm living on the prevalence of allergic rhinitis. In addition, they also found an increasing prevalence of allergic rhinitis with increasing degree of urbanization both in those raised on a farm and those who were not. Lin et al. [15] reported that adolescent asthma in Taiwan is most prevalent in the most urbanized areas and decreases in prevalence in less urbanized areas. From the literature review, there were no studies that mentioned urbanization and KD. Our study showed no association between KD and urbanization. Environmental factors may still have an impact on the distribution of KD. Rodó et al. [16] analyzed three major KD epidemics in Japan; the major nonepidemic interannual fluctuations of KD in Japan and San Diego; and the seasonal variations of KD in Japan, Hawaii, and San Diego. They revealed a consistent pattern wherein KD cases are often linked to large-scale wind currents originating in central Asia and traversing the north Pacific. The results suggest that wind-borne contaminants could be the key environmental factor for KD. Taiwan is located in a subtropical area, with very stable climate in four seasons. The climate in the four geographical locations (north, center, south, and east) of Taiwan is similar. 

The cause of Kawasaki disease is still unclear. Both infectious origins and genetic factors have been widely reported [1]. Although an infectious origin is highly suspected, the pathogen that triggers Kawasaki disease still has not been confirmed. Regarding genetic factors, single nucleotide polymorphisms in ITPKC, CASP3, and TGF-beta pathways were reported to be associated with the development of Kawasaki disease in a Taiwanese population [17–21]. Thus, genetic contributions may play a critical role in the development of Kawasaki disease. 

There are limitations to this study. Although urbanization was divided into seven levels based on Taiwanese census data, factors such as immigration rate, economic activities, educational level, average family income, and availability of health care facilities were not included. Our results indicated that most KD cases reside in higher urbanization levels such as northern Taiwan. This might be because study subjects in northern Taiwan were more likely to receive regular health checkups, possibly because of the prevalence of national medical centers that tend to be better equipped. Thus, diagnosis of KD would be much easier. 

In conclusion, we have explored the effects of factors associated with urbanization on the prevalence of Kawasaki disease. Our results provide evidence that urbanization is not associated with the prevalence of Kawasaki disease in Taiwan. To better explore this disease, future studies of the social mechanisms and lifestyles among rural and urban populations are required.

## Figures and Tables

**Table 1:** Demographic characteristics for selected KD patients, stratified by presence/absence of Kawasaki disease from 1997 to 2010 (*n* = 1265).

	Patients with Kawasaki disease (*n* = 115)		Patients without Kawasaki disease (*n* = 1150)		*P* value
	*N*	%	*N*	%	
Gender					1
Male	66	57.4	660	57.4	
Female	49	42.6	490	42.6	
Age (years)					1
0–2	86	74.8	860	74.8	
3–5	23	20.0	230	20.0	
6–8	6	5.2	60	5.2	

**Table 2:** Association between urbanization level and Kawasaki disease.

	Patients with Kawasaki disease (*n* = 115)		Patients without Kawasaki disease (*n* = 1150)		*P* value
	*N*	%	*N*	%	
Urbanization level					0.9
1 (most urbanized)	34	29.6	362	31.5	
2	29	25.2	286	24.9	
3	30	26.1	241	21.0	
4	14	12.2	154	13.4	
5	2	1.7	28	2.4	
6	3	2.6	36	3.1	
7 (least urbanized)	3	2.6	43	3.7	
Geographic region					0.2
North	64	55.7	544	47.3	
Center	21	18.3	312	27.1	
South	25	21.7	250	21.7	
East	5	4.3	44	3.8	
